# Impact of the Cognitive–Behavioral Approach and Psychoeducational Intervention in Breast Cancer Management: A Prospective Randomized Clinical Trial

**DOI:** 10.3390/healthcare10040629

**Published:** 2022-03-27

**Authors:** Antonella Ardizzone, Domenico Bavetta, Maria Luisa Garo, Domenico Santangelo, Antonio Bongiorno, Maria Bono

**Affiliations:** 1 U.O. Oncologia Medica, Hospital Giovanni Paolo II, 92019 Sciacca, Italy; posciacca.oncologia@aspag.it; 2 Samo Onlus, 92100 Agrigento, Italy; 3 Istituto Tolman Srl, 90121 Palermo, Italy; nellobongiorno@virgilio.it; 4 U.O. Ginecologia e Ostetricia Department, Hospital Giovanni Paolo II, 92019 Sciacca, Italy; domenicobavetta@libero.it; 5 Mathsly Research, 25100 Brescia, Italy; marilu.garo@gmail.com; 6 C.S.M.—Hospital Giovanni Paolo II, 92019 Sciacca, Italy; mariella.bono@libero.it

**Keywords:** breast cancer, cognitive behavioral approach, psychoeducational intervention, RCT

## Abstract

(1) Background: Breast cancer (BC) is the most prevalent malignancy in women. High cancer-related psychological distress levels have been observed in BC patients, with a potentially relevant impact on disease management, compliance with disease treatments, and everyday life activities and relationships. This work evaluated the effectiveness of three individual cognitive–behavioral therapy psychoeducational sessions versus a self-managed informative guide with individual counseling sessions without specific psychological treatment. (2) Methods: the intervention group received three individual 50-min sessions of psychoeducational training, and the control group received a self-managed informative guide with individual counseling sessions without any kind of psychological treatment. The Hospital Anxiety Depression Scale (HADS), the Distress Thermometer (DT), and the EORTC (European Organization for Research and Treatment of Cancer) QLQ-C30 were administered at baseline and two months after study inclusion. (3) Results: A total of 60 participants were included in the study (intervention group: 30, control group: 30). Significant improvements were observed in both groups after two months (*p* < 0.05), but no statistically significant differences emerged between groups. (4) Conclusions: Psychoeducational interventions and CBT help BC patients manage disease-related fear and distress, allowing them to achieve a good quality of life.

## 1. Introduction

Breast cancer accounts for 12% of all new annual cancer cases worldwide [1]. A total of 13.3% of new breast cancer cases were diagnosed in EU-27 countries, and a mortality rate of 7.2% was observed in 2020 [2]. According to US Breast Cancer Statistics, in 2022, 287,850 new cases of invasive breast cancer were diagnosed in the USA, of which 51,400 were new patients of non-invasive breast cancer [1]. In Europe, BC survival is approximately 80% [3], although this negative trend is steadily decreasing [4]. About 30–40% of breast cancer patients show various symptoms of emotional distress following diagnosis and treatments [5,6,7]. Cancer-related distress may be considered a continuum ranging from usual fears to severe anxiety or depression, leading to a progressive loss in the patients’ quality of life (QoL) [8]. A recent meta-analysis conducted by Sanjida et al. (2018), which evaluated the effects of psychological interventions in anxiety and distress reduction and analyzed the characteristics of the sample and the interventions that influenced effectiveness, showed that psychological interventions and supportive therapies reduce anxiety and distress [9]. On the other hand, unmet stress is correlated with poor adherence to treatment, poor QoL, and increased healthcare costs [10]. Therefore, the optimal management of psychological problems related to oncological disease should be determined by an accurate clinical evaluation, including pharmacological and non-pharmacological interventions [11].

Furthermore, providing information to patients and their family members or caregivers at all stages of the disease is one of the most critical elements of supportive care in oncology because adequate information helps patients reduce uncertainty and strengthen a sense of control, allowing them to make appropriate decisions [12]. In a systematic review aiming to evaluate the effectiveness of interventions carried out by specialist breast care nurses regarding the QoL of patients with breast cancer, positive effects between the early recognition of depressive symptoms and some components of QoL were observed [13]. Early assessment and implementation of treatments to reduce distress are necessary to increase treatment adherence, reduce visits and admissions, and improve psychological wellbeing [14]. In Italy, despite the presence of new treatment protocols, the AIOM (Associazione Italiana di Oncologia Medica) and Sipo (Italian Society of Psycho-oncology) guidelines (2019) have highlighted that the need for information is among the most relevant psychological needs of patients suffering from neoplastic pathology [15]. Furthermore, a study on women with breast cancer showed that the need for more information about the disease and psychological problems related to the disease were prevalent and intense requests (70%) [16].

In the field of psycho-oncology, four macro-categories of intervention are possible: counseling, behavioral methods, bodily methods, and psychotherapies [17], among which psychoeducational and cognitive–behavioral approaches have shown significant efficacy [18]. Indeed, as noted by the research conducted by Lally et al. (2019) on the U.S. population, psychoeducational treatments may improve patients’ quality of life because psychological approaches provided in the form of individual or group psychoeducation or through informative booklets or guides increase patients’ disease awareness and reduce distress levels, thus positively influencing adherence to treatments through an increase in disease acceptance and a path to learn about how to manage the negative aspects of the disease and related therapies [19]. In addition, a recent meta-analysis showed that cognitive–behavioral therapy (CBT)—a psychological intervention aimed at improving mental health [20] that combines the basic principles of behavioral and cognitive psychology [21]—showed a positive effect on breast cancer patients for the treatment of mental disorders [22]. Indeed, CBT has a positive action on improving anxiety in BC patients. Still, there is no evidence of positive impacts in reducing depression symptoms and improving quality of life [21].

This work aimed to evaluate the effect of two psychological approaches in increasing the quality of life level and reducing depression, anxiety, and distress in two groups of women with breast cancer who underwent two different approaches: three CBT individual psychoeducational sessions versus a self-managed informative guide with individual counseling sessions without any specific psychological treatment.

## 2. Materials and Methods

### 2.1. Study Design

A monocentric prospective randomized clinical study was carried out at the U.O. of Medical Oncology of the P.O. “Giovanni Paolo II” of Sciacca (Italy) from October 2020 to March 2021. The Ethical Committee of Palermo approved the study (date of approval: 14 September 2020, protocol approval: 155/A.G.).

### 2.2. Participants

Adult women who have breast cancer, treated with chemo- or radiotherapy and who had already undergone surgery, afferent to the U.O. of Medical Oncology of the P.O. “Giovanni Paolo II” of Sciacca, were selected to participate in the study. The medical staff consisted of an oncologist and a psychotherapist. Patients were enrolled according to the following inclusion criteria: (1) aged between 35–70 years; (2) patients with breast cancer undergoing treatment (radiotherapy, chemotherapy, or hormone therapy) and undergoing surgery (mastectomy, quadrantectomy, or similar). The exclusion criteria were: (1) patients with psychiatric pathologies as detected through the Symptom Checklist-90 Scale (SCL-90-R) and/or medical records; (2) patients suffering from secondary metastases or neoplastic pathologies (head–neck, colorectal, intestine, or ovary); (3) patients suffering from blood cancers and sarcomas. Sixty patients were enrolled and assigned to the intervention or control group, respectively. All the participants signed informed consent forms.

The intervention group received three individual 50-min session of psychoeducational training, and the control group received a self-managed informative guide with individual counseling sessions without any kind of psychological treatment.

According to a 1:1 randomization process, the participants were randomized into two groups. The clinicians randomly generated the treatment allocations using sealed opaque envelopes and assigned these to the patients accordingly. The researchers and clinicians knew to which of both of the groups the individuals belonged. Only the patients were blinded.

The Hospital Anxiety Depression Scale (HADS) [23], the Distress Thermometer (DT) [14,24], and the EORTC QLQ-C30 (Italian version 3.0) [25] were administered at baseline and after two months from inclusion in the study.

### 2.3. Sample Size

The sample size was evaluated according to two previous studies [26]. The sample size was determined using the following parameters: *α* = 0.05, 1 − *β* = 0.8, and d = 0.74 (G * Power, Heinrich Heine University of Düsseldorf, Düsseldorf, Germany). As a result, the required sample size was determined to be 60 (treatment group: 30; control group: 30).

### 2.4. Procedures

#### CBT Psychoeducational Intervention

The theoretical model of reference was the cognitive–behavioral approach: an active treatment focused on the problems presented by the patient at the time of the interview and limited in time. It is based on cooperation and transparency. The patient’s expertise regarding their living condition is as necessary as the therapist’s expertise regarding the disease and the cognitive–behavioral approach [27].

In the present study, psychoeducational techniques were used to develop correct information on the disease, favoring both the acceptance and processing of the diagnosis itself and the recognition, understanding, and management of the pathology [28].

The CBT psychoeducational intervention consisted of three individual meetings lasting 50 min, aiming to deepen the following topics: (1) breast cancer types, symptoms, risk factors, diagnosis, and importance of prevention; (2) surgical treatment and chemo-radiotherapy; and (3) the emotional and psychological impact of the course of care and the use of functional strategies.

### 2.5. Counseling Sessions

Patients received an informative guide containing clinical information, psychological aspects, and indications related to breast cancer. This guide was developed using information reported by the AIMAC (https://www.aimac.it, accessed on 10 November 2020), ESMO (https://www.esmo.org/for-patients/patient-guides, accessed on 10 November 2020), and AIRC (https://www.airc.it/cancro, accessed on 10 November 2020) guides. The main aim of this guide was to accompany patients and their caregivers in the care path by providing adequate information. The first part of the guide contained topics concerning breast cancer, breast cancer histotypes, the staging and evolution of the disease, symptoms, diagnosis, and risk factors (including heredity, familiarity, and BRCA-1 and BRCA-2 genetic mutations). The following section included information on the various forms of treatment for breast cancer, such as surgery, radiotherapy, hormone therapy, molecular target therapy, and chemotherapy. In addition, there were indications and precautions to help reduce chemotherapy side effects to improve the patients’ quality of life after breast cancer surgery. The last part of the guide contained aspects related to the follow-up after the treatment, lifestyles after surgery, secondary prevention, and preventive diets. In addition, this section included helpful links, support groups, and information on the patients’ rights. Finally, a glossary containing 26 terms from the medical and psychological fields was used to help patients understand some terms often used by oncologists during a specialist medical interview. Given the complexity of the disease and the breast cancer impact on the patient’s psychological sphere, patients in the control group received individual counseling and psychological listening meetings without specific treatments.

### 2.6. Statistical Analysis

After HADS, DIS, and EORTC QLQ-C30 data collection (Excel file, Microsoft Corp., Redmond, WA, USA), statistical analyses were performed using STATA17 (StataCorp., College Station, TX, USA). Quantitative variables were reported as mean ± standard deviation, while frequencies and percentages were used for qualitative variables. Fisher’s exact test, the Mann–Whitney U test, and the Wilcoxon test on paired samples were used for group and intra-group comparisons. Significance was set at 5% (*p* < 0.05).

## 3. Results

**Figure 1:**
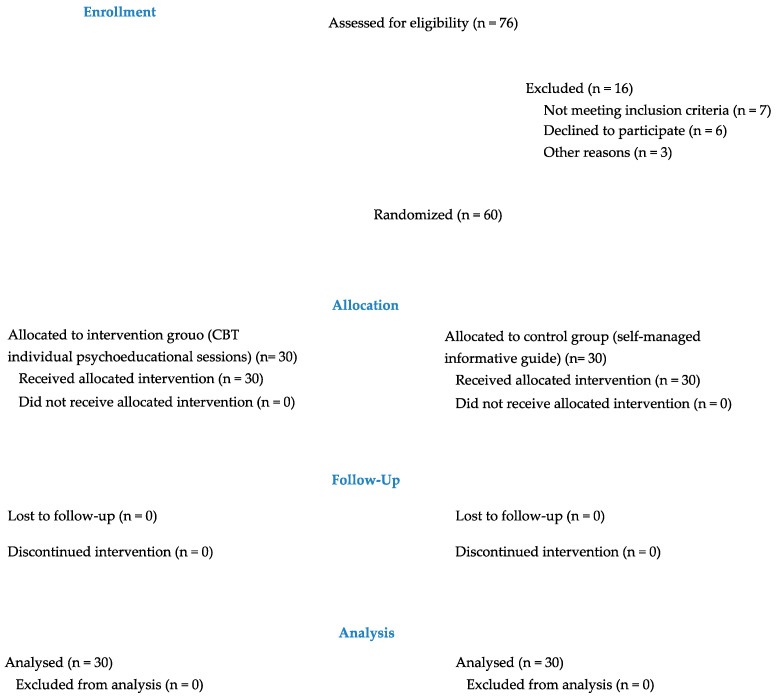
CONSORT Flow-chart.

A total of 60 participants were included in the study and randomly assigned to groups (treatment group: 30; control group: 30). All the enrolled patients completed the study and were included in the analysis as reported by the CONSORT flow chart (Figure 1).

About 43% (*n* = 26) of the women were aged between 51 and 65 years, 38.3% (*n* = 23) over 65 years old, and 18.3% (*n* = 11) between 36 and 50 years old. More than 73% (*n* = 44) of the women had received a BC diagnosis two to three years before enrollment. Many participants (73.3%, *n* = 44) had received neoadjuvant chemotherapy before surgery; 31.7% (*n* = 19) had received postmenopausal hormone treatment; 31.7% (*n* = 19) had received radiotherapy, and a similar percentage had been treated with biological therapy; while 30% (*n* = 18) of the participants had received molecular target therapy. At baseline, no statistically significant differences emerged. The participants’ characteristics are reported in Table 1.

**Figure 2:**
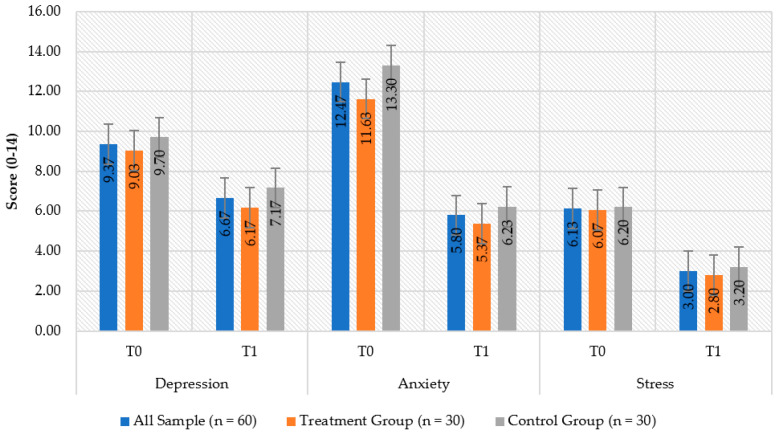
Depression, anxiety and distress in the intervention and control groups at baseline (T_0_) and after two months (T_1_).

No statistical differences emerged when comparing the treatment and control groups at time T1 (*p* > 0.05) for HADS and EORT QLQ-C30. Instead, many improvements were registered in both groups between baseline and T1. Specifically, in HADS, statistically significant differences between baseline and T1 emerged for depression (treatment_T0_ = 9.03 ± 3.36, treatment_T1_ = 6.17 ± 2.67, *p* < 0.001; control_T0_ = 9.70 ± 2.69, control_T1_ = 7.17 ± 2.44, *p* < 0.001), anxiety (treatment_T0_ = 11.63 ± 4.43, treatment_T1_ = 5.37 ± 2.95, *p* < 0.001; control_T0_ = 13.30 ± 3.50, control_T1_ = 6.23 ± 2.22, *p* < 0.001), and stress (treatment_T0_ = 6.07 ± 1.20, treatment_T1_ = 2.80 ± 1.03, *p* < 0.001; control_T0_ = 6.20 ± 1.03, control_T1_ = 3.20 ± 0.96, *p* < 0.001) (Figure 2).

**Figure 3:**
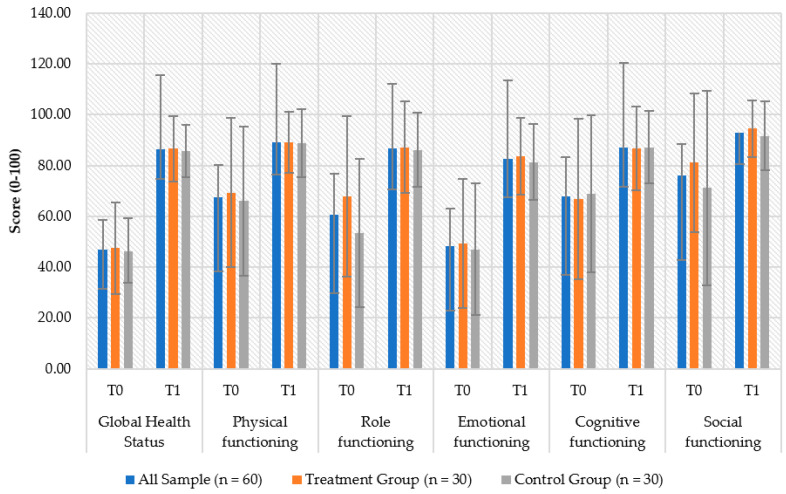
EORTC-QLQ30.

According to the EORTC QOL-C30 scale, improvements were recorded in both groups between T0 and T1 for global health status (treatment_T0_ = 47.50 ± 18.07, treatment_T1_ = 86.67 ± 12.87, *p* < 0.001; control_T0_ = 46.39 ± 12.70, control_T1_ = 88.89 ± 13.37, *p* < 0.001), physical functioning (treatment_T0_ = 69.33 ± 29.38, treatment_T1_ = 89.11 ± 11.94, *p* < 0.001; control_T0_ = 66.00 ± 29.42, control_T1_ = 88.89 ± 13.37, *p* < 0.001), role functioning (treatment_T0_ = 67.78 ± 31.54, treatment_T1_ = 87.22 ± 17.88, *p* < 0.001; control_T0_ = 53.33 ± 29.16, control_T1_ = 86.11 ± 14.57, *p* < 0.001), emotional functioning (treatment_T0_ = 49.44 ± 25.42, treatment_T1_ = 83.61 ± 15.08, *p* < 0.001; control_T0_ = 46.94 ± 25.94, control_T1_ = 81.39 ± 14.95, *p* < 0.001), cognitive functioning (treatment_T0_ = 66.67 ± 31.56, treatment_T1_ = 86.67 ± 16.61, *p* < 0.001; control_T0_ = 68.89 ± 30.87, control_T1_ = 87.22 ± 14.31, *p* < 0.001), and social functioning (treatment_T0_ = 81.11 ± 27.24, treatment_T1_ = 94.44 ± 11.01, *p* < 0.001; control_T0_ = 71.11 ± 38.14, control_T1_ = 91.67 ± 13.67, *p* < 0.001) (Figure 3).

According to the QLQ-C30 EORTC symptom scales (Table 2), significant symptom reductions emerged in both groups between baseline and T1 for fatigue (treatment_T0_ = 35.56 ± 24.13, treatment_T1_ = 12.59 ± 12.29, *p* < 0.001; control_T0_ = 46.30 ± 22.81, control_T1_ = 14.44 ± 11.36, *p* < 0.001), nausea and vomiting (treatment_T0_ = 16.67 ± 30.01, treatment_T1_ = 4.44 ± 10.66, *p* = 0.006; control_T0_ = 26.11 ± 34.38, control_T1_ = 6.11 ± 10.25, *p* < 0.001), pain (treatment _T0_ = 36.11 ± 35.85, treatment_T1_ = 14.44 ± 13.66, *p* < 0.001; control_T0_ = 46.11 ± 29.26, control_T1_ = 17.22 ± 11.97, *p* < 0.001), dyspnea (treatment_T0_ = 22.22 ± 35.38, treatment_T1_ = 7.78 ± 16.80, *p* = 0.004; control_T0_ = 28.89 ± 34.72, control_T1_ = 7.78 ± 14.34, *p* < 0.001), and insomnia (treatment_T0_ = 46.67 ± 34.57, treatment_T1_ = 15.56 ± 16.91, *p* < 0.001; control_T0_ = 56.67 ± 27.89, control_T1_ = 21.11 ± 16.34, *p* < 0.001). Moreover, in the control group, significant improvement was observed for appetite loss (control_T0_ = 33.33 ± 36.09, control_T1_ = 7.78 ± 14.34, *p* < 0.001).

## 4. Discussion

Supporting patients and their families or caregivers to cope with and adapt to the difficulties associated with BC plays an overarching role in managing the disease and the treatment-related side effects. Therefore, this study aimed to evaluate the effectiveness of cognitive–behavioral therapeutic intervention, specifically psychoeducation, to improve the quality-of-life levels and to reduce depression, anxiety, and stress.

Being informed is reported as a key element in managing breast cancer, but, to date, it is mistakenly believed that patients prefer not to know (a culture of “non-disclosure”). A recent survey found that, contrary to what one might expect, the average number of questions asked during the first visit with the oncologist by Italian women with BC was higher as compared with the number of questions asked by patients in other countries [29].

In our work, all the participants reported a significant improvement in the pre–post comparisons without statistically significant differences between groups. Overall, 65% of the women at baseline showed an elevated anxiety level (≥11), while only 8.33% of the participants continued to report the same high anxiety level after two months. A similar trend emerged for depression: while 18% of the participants reported severe depression symptoms at enrollment, only 3.33% of the women continued to report severe depressive symptoms in the follow-up phase. Finally, encouraging results emerged for distress: 36.6% of the participants showed a high degree of distress (DT > 7) at enrollment, but none showed distress in any form after two months.

These findings for distress reduction confirm the positive results that have already emerged about a strong association between psychological interventions and the reduction of distress [30,31,32], and they showed a similar trend to that in a recent prospective randomized clinical study, which compared the efficacy of an innovative guide (CaringGuidance^TM^ program, University of Nebraska Medical Center, Omaha NE, USA) with that achieved through the usual care after 12 weeks of treatment: a reduction in distress and the avoidance of negative thoughts without reporting significant differences in the overall effects between the two groups was observed in both groups [19]. For improvement in distress symptoms reduction, Jacobsen et al. (2002), analyzing the impact of a self-administered CBT-based stress management intervention in a heterogeneous cancer population, showed a significant decrease in distress levels for the intervention group compared to the control group who did not receive any kind of psycho-intervention programs [33]. Conflicting results have emerged about the ability of CBT workbooks to reduce distress; according to Krisher et al. (2007), these self-managed interventions may reduce baseline distress [34], while according to Angell et al. (2003), self-help workbooks are efficient in reducing distress in patients who completed treatment. Still, they do not affect those with a recent diagnosis [35]. The positive role of psychological intervention was already confirmed in recent meta-analyses. According to Sanjida et al. (2018), who conducted a meta-analysis including 51 RCT studies comprising a total of 13,098 patients, the overall effect size of psychological interventions (CBT, psychosocial therapy, and relaxation) for anxiety reduction was −0.21 (95% CI −0.30 to −0.13); more significant effect sizes in the subgroup analysis were obtained for relaxation training (number of studies = 12; effect size: −0.53), individual mode (n. studies = 24; effect size: −0.32), and face to face with self-administration (number of studies = 7; effect size: −0.35) [9]. Another meta-analysis of 41 RCT studies carried out by Coutiño-Escamilla et al. (2019), comprising a total of 4869 patients and evaluating the impact of non-pharmacological interventions in reducing depressive symptoms in patients with breast cancer, showed a relevant reduction in depressive symptoms (standardized mean difference, SMD = −0.516, 95%CI −0.814 to −0.218), with a considerable statistically significant impact of psychotherapy (SMD = −0.819, 95% CI −1.608 to −0.030, *p* = 0.042) and yoga (SMD = −0.385, 95%CI −0.633 to −0.136, *p* = 0.002) [36].

All the patients in our study reported significant improvements in quality of life and symptom reduction in relation to quality of life. Still, no statistically significant differences emerged between the two groups. These effects show how informative guides and psychological supports have benefited both groups, allowing them to achieve a general psychological, social, and relational wellbeing. These findings are in line with those that emerged in similar studies in which, for ethical considerations, the control group was treated with a form of external support. A recent study analyzing the role of psychoeducational programs focused on psychological stresses and management skills in a sample of 290 BC patients during radiotherapy did not only report statistically significant differences between the intervention and control group, as also emerged in our work, but the control group, who received a 15-min educational course on basic knowledge of breast cancer and radiotherapy, also showed an increase in anxiety and depression levels [37]. For CBT, the reasons for the lack of significant differences could be explained in light of a previous study that showed a substantial positive impact of CBT in improving anxiety symptoms, but no action in improving depression and quality of life [21,38].

Moreover, it is necessary to consider the environmental context in which this study was conducted (October 2020 to March 2021). In Italy, during the last two years, the prevalence of the COVID-19 pandemic could have determined a different sense of the quality of life and distress, especially in oncologic patients, who, even owing to few external supports, such as an informative and complete guide, could have achieved an optimal quality of life perception. Furthermore, the individual counseling meetings, during which participants could have had the opportunity to discuss some topics reported in the informative guide, and the lack of group therapy for the intervention group, which was not allowed given the stringent regulations about COVID-19 containment, could have further reduced the differences between the two groups. Indeed, as emerged in previous studies, group therapy represents a fundamental factor in creating affective–relational and informative interrelations that facilitate the perception of the disease as an opportunity for a significant existential improvement and provide a psychological sense of belonging to the group, a possible way to manage stress and overcome the sense of isolation [17,38]. Cipolletta et al. (2019) showed that group psychoeducation reduces anxiety, depression, and fatigue in BC patients and increases their adaptability to the same disease, creating a sense of being able to control the cancer and giving the patients space to improve their emotional functioning and enlarge their interpersonal relationships [17].

As with other trials, this work has several limitations. First, although we used validated tools to assess anxiety, distress, depression, and quality of life, few studies have discussed the diagnostic role of these tools. As already emerged in other works, conclusions of no statistically significant differences between the two groups should not be considered conclusive. Second, this study was conducted during the largest pandemic in the last 100 years in Europe; this means that participants’ perception could have been modified because of a different approach: a significant threat from the COVID-19 pandemic and less attention to one’s own distress level. Third, we enrolled patients with various comorbidities and times of diagnosis. Fourth, the observation time and the number of CBT sessions could have been too short or too few. Five, a Hawthorne effect could have changed the patients’ behavior and responses. Finally, we did not consider a control group without psychological intervention.

## 5. Conclusions

In conclusion, our understanding of the different impacts of CBT and psychoeducation could be deepened in future studies. However, from this work, we can affirm that both psychoeducational intervention and CBT can help patients with BC to overcome the fear of the disease and to achieve an optimal distress level management and a good quality of life because these psychological supports provide a source of information about their condition and reduce the sense of isolation.

## Figures and Tables

**Table 1:** Participants characteristics.

	Whole Sample (*n* = 60)	Treatment Group (*n* = 30)	Control Group (*n* = 30)	*p*-Value
Age				
*36–50 years*	11 (18.33%)	7 (23.33%)	4 (13.33)	0.190
*51–65 years*	26 (43.33%)	15 (50.00%)	11 (36.67)	
*>65 years*	23 (38.33%)	8 (26.67%)	15 (50.00)	
Time from the first diagnosis				
*1–3 years*	49 (81.7%)	23 (76.7%)	26 (86.7%)	0.506
*>3 years*	11 (18.3%)	7 (23.3%)	4 (13.3%)	
Qualification				
*High School*	57 (95.0%)	28 (93.3%)	29 (96.7%)	1.000
*Degree or Higher*	3 (5.0%)	2 (6.7%)	1 (3.3%)	
Marital Status				
*Single*	5 (8.33%)	3 (10.00%)	2 (6.67%)	0.229
*Married*	46 (76.67%)	25 (83.33%)	21 (70.00%)	
*Divorced*	0 (0.00%)	0 (0.00%)	0 (0.00%)	
*Widower*	9 (15.00%)	2 (6.67%)	7 (23.33%)	
BMI				
*Underweight (≤18.49)*	1 (1.67%)	1 (3.33%)	0 (0.00%)	0.482
*Normal weight (18.5–24.99)*	19 (31.67%)	11 (36.67%)	8 (26.67%)	
*Overweight (25.0–29.99)*	29 (48.33%)	12 (40.00%)	17 (56.67%)	
*Obese (≥30)*	11 (18.33%)	6 (20.00%)	5 (16.67%)	
Diabetes	5 (8.33%)	2 (6.67%)	3 (10.00%)	1.000
Use of antidepressants or benzodiazepines	3 (5.00%)	2 (6.67%)	1 (3.33%)	1.000
Psychiatric disease	0 (0.00%)	0 (0.00%)	0 (0.00%)	NA
Staging				
*0*	0 (0.00%)	0 (0.00%)	0 (0.00%)	0.923
*I*	10 (16.67%)	4 (13.33%)	6 (20.00%)	
*II*	24 (40.00%)	13 (43.33%)	11 (36.67%)	
*III*	12 (20.00%)	6 (20.00%)	6 (20.00%)	
*IV*	14 (23.33%)	7 (23.33%)	7 (23.33%)	
Histologic grade				
*Grade 1*	4 (6.67%)	1 (3.33%)	3 (10.00%)	0.475
*Grade 2*	24 (40.00%)	11 (36.67%)	13 (43.33%)	
*Grade 3*	32 (53.33%)	18 (60.00%)	14 (46.67%)	
Surgical treatment	37 (61.67%)	19 (63.33%)	18 (60.00%)	1.000
Reconstruction	0 (0.00%)	0 (0.00%)	0 (0.00%)	NA
Chemotherapy				
*None*	5 (8.33%)	2 (6.67%)	3 (10.00%)	1.000
*Adjuvant*	11 (18.33%)	6 (20.00%)	5 (16.67%)	
*Neoadjuvant*	44 (73.33%)	22 (73.33%)	22 (73.33%)	
Hormonal treatment				
*None*	36 (60.00%)	16 (53.33%)	20 (66.67%)	0.614
*Pre-menopause*	5 (8.33%)	3 (10.00%)	2 (6.67%)	
*Post-menopause*	19 (31.67%)	11 (36.67%)	8 (26.67%)	
Radiotherapy	19 (31.67%)	9 (30.00%)	10 (33.33%)	1.000
Biological treatment	19 (31.67%)	10 (33.33%)	9 (30.00%)	1.000
Molecular targeted treatment	18 (30.00%)	9 (30.00%)	9 (30.00%)	1.000
Lymphedema	10 (16.67%)	5 (16.67%)	5 (16.67%)	1.000

**Table 2:** EORTC QLQ-C30 Symptoms.

		Whole Sample (*n* = 60)		Treatment Group (*n* = 30)		Control Group (*n* = 30)		*p*-Value
Fatigue	T_0_	40.93	±23.90	35.56	±24.13	46.30	±22.81	0.117
	T_1_	13.52	±11.77	12.59	±12.29	14.44	±11.36	0.467
	*p*-value	0.000		0.000		0.000		
Nausea and Vomiting	T_0_	21.39	±32.34	16.67	±30.01	26.11	±34.38	0.214
	T_1_	5.28	±10.40	4.44	±10.66	6.11	±10.25	0.355
	*p*-value	0.000		0.006		0.000		
Pain	T_0_	41.11	±32.83	36.11	±35.85	46.11	±29.26	0.137
	T_1_	15.83	±12.81	14.44	±14.66	17.22	±11.97	0.355
	*p*-value	0.000		0.000		0.000		
Dyspnea	T_0_	25.56	±34.92	22.22	±35.38	28.89	±34.72	0.325
	T_1_	7.78	±15.49	7.78	±16.80	7.78	±14.34	1.000
	*p*-value	0.000		0.004		0.000		
Insomnia	T_0_	51.67	±31.55	46.67	±34.57	56.67	±27.89	0.274
	T_1_	18.33	±16.72	15.56	±16.91	21.11	±16.34	0.299
	*p*-value	0.000		0.000		0.000		
Appetite Loss	T_0_	23.89	±33.67	14.44	±28.61	33.33	±36.09	0.024
	T_1_	6.67	±13.45	5.56	±12.63	7.78	±14.34	0.748
	*p*-value	0.000		0.055		0.000		
Constipation	T_0_	13.89	±27.65	12.22	±26.96	15.56	±28.68	0.638
	T_1_	4.44	±11.43	4.44	±1.52	4.44	±11.52	1.000
	*p*-value	0.001		0.125		0.016		
Diarrhea	T_0_	7.78	±18.78	5.56	±17.69	10.00	±19.87	0.292
	T_1_	2.78	±9.29	1.11	±6.09	4.44	±11.52	0.353
	*p*-value	0.008		0.250		0.063		
Financial Difficulties	T_0_	4.44	±15.61	3.33	±13.42	5.56	±17.69	0.806
	T_1_	0.56	±4.30	1.11	±6.09	0.00	±0.00	1.000
	*p*-value	0.063		0.500		0.250		

## Data Availability

Not applicable.
